# GliomaPredict: a clinically useful tool for assigning glioma patients to specific molecular subtypes

**DOI:** 10.1186/1472-6947-10-38

**Published:** 2010-07-15

**Authors:** Aiguo Li, Serdar Bozdag, Yuri Kotliarov, Howard A Fine

**Affiliations:** 1 Neuro-Oncology Branch, National Cancer Institute, National Institutes of Neurological Disorder and Stroke, National Institutes of Health, Bethesda, MD 20892, USA

## Abstract

**Background:**

Advances in generating genome-wide gene expression data have accelerated the development of molecular-based tumor classification systems. Tools that allow the translation of such molecular classification schemas from research into clinical applications are still missing in the emerging era of personalized medicine.

**Results:**

We developed GliomaPredict as a computational tool that allows the fast and reliable classification of glioma patients into one of six previously published stratified subtypes based on sets of extensively validated classifiers derived from hundreds of glioma transcriptomic profiles. Our tool utilizes a principle component analysis (PCA)-based approach to generate a visual representation of the analyses, quantifies the confidence of the underlying subtype assessment and presents results as a printable PDF file. GliomaPredict tool is implemented as a plugin application for the widely-used GenePattern framework.

**Conclusions:**

GliomaPredict provides a user-friendly, clinically applicable novel platform for instantly assigning gene expression-based subtype in patients with gliomas thereby aiding in clinical trial design and therapeutic decision-making. Implemented as a user-friendly diagnostic tool, we expect that in time GliomaPredict, and tools like it, will become routinely used in translational/clinical research and in the clinical care of patients with gliomas.

## Background

Gliomas are a family of malignant primary brain tumors found in adults and children and are associated with a high motility. Gliomas have historically been pathologically categorized based on their presumed histological origin (i.e. astrocytic, oligodendroglial), as determined by standard light microscopy-based cellular morphology [1]. These broad categories are further subdivided into the "grade" of the tumor, a representation of the predicted relative aggressiveness of the tumor as determined by other histology-based variables (i.e. necrosis, endothelial proliferation, nuclear pleomorphism) [2]. While the histopathological approach is still the standard utilized in the clinic, it is well recognized that such classifications are limited by significant intraobserver variability, the lack of a biological basis for the designation, and the relatively poor prognostic or therapeutic predictive utility of the system [3,4]. A more objective, reproducible and biologically meaningful classification system is clearly needed.

The molecular stratification of glioma patients into subtypes is the most direct way to translate genome-wide high-throughput data into information that can be used in a clinical setting. In the past, several groups have attempted to identify brain tumor subtypes computationally and stratify glioma patients into subtypes in terms of the gene expression pattern of their classifiers. Limited sample size (11-84 samples) and the subjective selection of gene features, however, impaired the robustness and clinical application of these classification methods [5-8]. Recently, we established a robust and reliable glioma classification system that includes six hierarchically-nested subtypes and six sets of classifiers. Derived from transcriptomic profiles of 159 glioma patients [9], samples are separated into two main types: oligodendrogliomas enriched (O) and glioblastomas enriched (G). While the O group was further divided into OA and OB subtypes, the G group was split into four subtypes, showing hierarchically-nested relationship designated as GA1, GA2, GB1 and GB2. Several recent studies, using other large and different databases, have likewise confirmed the existence of four subgroups within the glioblastoma designation. Whether the four subgroups identified by these different studies are all the same is not formally known at this time, however, we have found significant overlap between our four glioblastoma-enriched subtypes and the four subtypes identified in two of the largest classification studies of high-grade gliomas published to date (data not shown) [10,11]. To translate our glioma classification system into a clinically useful application that is readily usable by clinicians and other statistically and computationally naïve users, we implemented GliomaPredict, a computational tool that allows anyone to reliably predict the subtype of a new glioma patient utilizing his/her tumor gene expression profile. As an output, results are summarized in a printable PDF-file.

## Implementation

### Design objectives and specifications

We aimed to develop and implement a tool (GliomaPredict) that could easily be used by physicians and clinicians, as well as laboratory researchers, allowing the fast assignment of new patients to our previously-derived six glioma subtypes with minimal technical difficulties and user interventions. To ensure simplicity and eliminate user intervention, all the data transfer steps in GliomaPredict are handled by perl scripts. The navigation interfaces are simple and intuitive and the output is presented both quantitatively and graphically to enhance the visibility. Most importantly, we took the user-centered approach, designing the GliomaPredict as a plugin application of GenePattern, a powerful genomic analysis framewok that provides easy access to multiple applications for gene expression and SNP data analysis through a web-based interface [12]. Data formats and the abundance of other applications in GenePattern potentially allow a further integrative analysis of patient data in the future, combining data as disparate as genomic alterations, ChIP-ChIP and protein-protein interactions.

### Algorithm for assigning new patients to a subtype

GliomaPredict facilitates a supervised PCA using predefined gene classifiers to assign new patients to the six glioma subtypes [13]. The original algorithm was implemented in MATLAB (utilizing PRINCOMP function in Statistics Toolbox) that can be mathematically described as A = *U*∑V^*t*^, where *A *is a data matrix, *U *is the basis eigenvector and *V*^*t *^is the transposed matrix of all eigenvectors. The PCA sequentially selects new orthogonal coordinates (columns of *U*) capturing as much of the remaining variation in the data as possible to effectively reduce the dimensions of the data.

### Algorithm for assessing prediction reliability

We computed the probability that an unknown sample belongs to a certain glioma subtype using a supervised PCA of gene classifiers; the PCA projects the unknown sample to a *n*-dimensional space of reference dataset. The proportion of variation of PCA components is used as a weight vector to adjust distances. The weighted Euclidian distance between two points *U *= (*u*_*1*_, *u*_*2*_,..., *u*_*n*_) and *V *= (*v*_*1*_, *v*_*2*_,..., *v*_*n*_) in a *n-*dimensional space is defined as $d\left(U,V\right)=\sqrt{\sum_{i=1}^{n}w_{i}{\left(u_{i}-v_{i}\right)}^{2}}$, where *w *is the weight vector we obtained from PCA analysis. Based on the weighted Euclidian distance of unknown sample to the centroid of the training samples in the reference set that belong to a certain subtype we calculated the probability that an unknown sample *U *belongs to a given subtype *C*_*i *_as $p\left(U,C_{i}\right)=d{\left(U,{\bar{C}}_{i}\right)}^{-1}/\sum_{i=1}^{m}d{\left(U,{\bar{C}}_{i}\right)}^{-1}$, where *m *is the number of clusters, and ${\bar{C}}_{i}$ is the centroid of samples in subtype *C*_*i*_.

Computing the relative distance of unknown samples to other subtypes, we defined the relative distance as a measure of a sample being an outlier. In particular, the relative distance *r*_*D *_of an unknown sample *U *to a subtype *C*_*i *_is computed as $r_{D}(U,C_{i})=d\left(U,{\bar{C}}_{i}\right)/\sigma \left(\underset{s\in C_{i}}{d\left(s,{\bar{C}}_{i}\right)}\right)$, where σ is the standard deviation of distances between the samples of a subtype *C*_*i *_to the corresponding centroid ${\bar{C}}_{i}$.

### Installation

MATLAB version 7a or higher with Statistical Toolbox, Perl version 5.8 or higher and GenePattern server version 3.0 or higher http://genepattern.org must be installed on the server side. Acrobat viewer should be installed on the client terminal for viewing the PDF-file, summarizing the output. GliomaPredict was tested using Internet Explorer (ver. 6.0 or higher) or Firefox (ver. 3.0 for Mac & PC or higher) and Safari (ver. 3.2 or higher) (Additional file 1, Additional file 2 and Additional file 3).

## Results

### Overview of GliomaPredict

**Figure 1:**
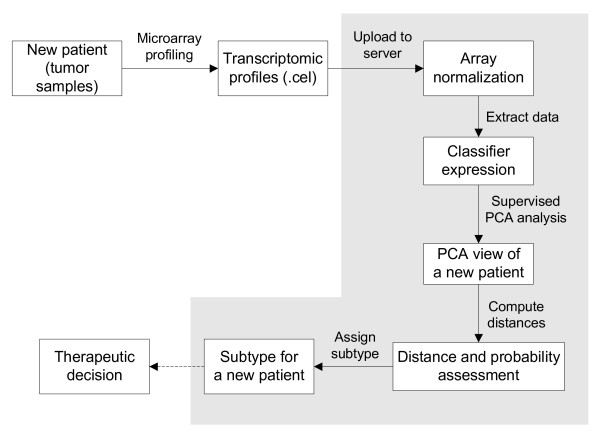
**Step-wise workflow of the GliomaPredict tool**. Shaded area refers to functions and features included in the GliomaPredict tool. Specifically, our tool demands a transcriptomic .cel file of an unknown patient sample as input; subsequently .cel file is automatically normalized. After filtering classifier-specific values, GliomaPredict performs a supervised PCA analysis, provides a graphical representation of the PCA results and returns the quantitative measures that assess the reliability of the underlying patient-designated classification.

We implemented GliomaPredict as a web-based application that assigns a new patient to one of six predefined glioma subtypes using three sets of classifiers. The prediction is performed by running a supervised PCA analysis using classifier-based gene expression in a reference set. The prediction probability and distances are computed from the output of PCA analysis, and the new patient is accordingly assigned to a subtype (Figure 1). Using the projected mapping of the reference set classifiers to discriminate between glioma subtypes, GliomaPredict offers a flexible solution, allowing users to utilize their laboratory and/or microarray platform specific sets of gene classifiers.

#### Data requirements, workflow and output

GliomaPredict depends on a reference dataset to assign a new patient to a particular tumor subtype. Therefore, the reference dataset needs to be uploaded to the server during installation. It takes three steps to create a reference dataset: (i) profiling a set of glioma tumor arrays using Affymetrix HG-U133A, HG-U133AB or HG-U133 plus 2 platforms, (ii) stratifying the arrays into the major subtypes (six subtypes in our classification) using classifiers [9], (iii) create a reference dataset file in MATLAB structure format that contains classifier expression of O/G, OA/OB and GA1/GA2/GB1/GB2 subtypes. The details for creating a reference set are available in the GliomaPredict manual. In addition, expression .cel files of new glioma patients must be uploaded to a server before the prediction analysis is run (Additional file 1, Additional file 2). The .cel files will be normalized automatically after uploading and a .txt file containing normalized expression values is created and stored into GliomaPredict tool. A user can then select the new patient from the patient pulldown list for prediction. While a user chooses an array for classification, the classifier expression of that patient is extracted and merged into the reference dataset.

**Figure 2:**
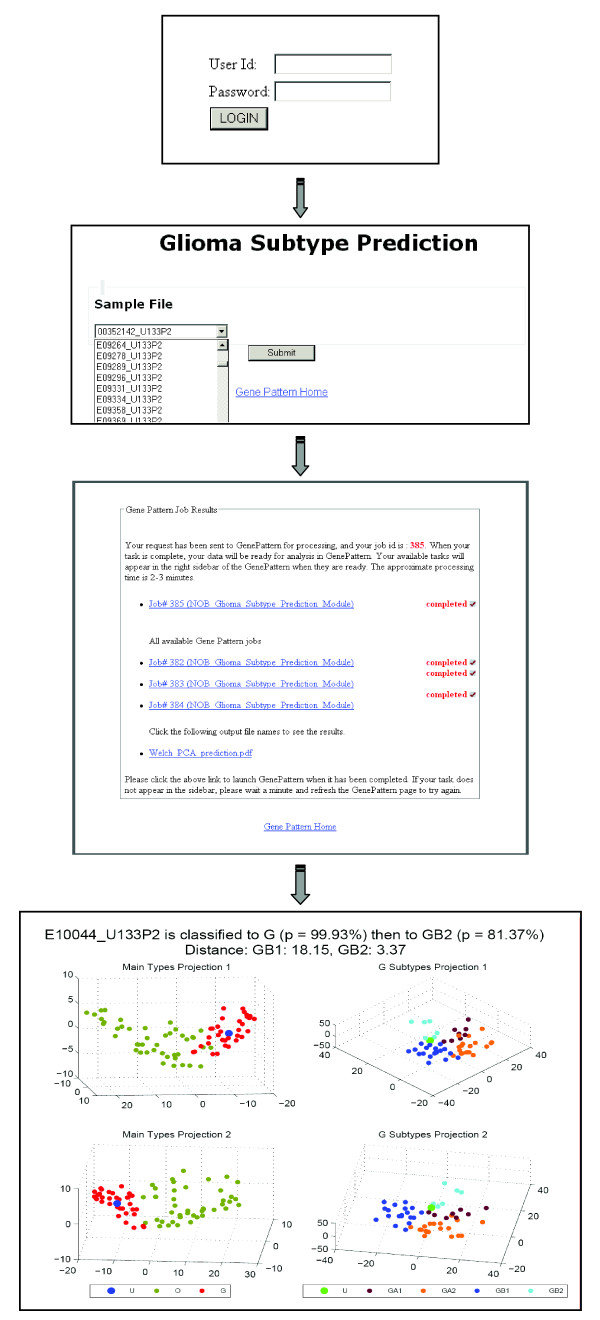
**User interfaces, prediction process and output examples of the GliomaPredict tool**. Predicting the subtype of an unknown patient sample, GliomaPredict provides a visual representation of the PCA of all reference samples in the subgroups. Indicating the location of an unknown patient sample in these data clouds, GliomaPredict calculates a probability that the underlying sample is of a certain subgroup. In addition, GliomaPredict also accounts for a distance of the underlying sample to the centroid of the subgroups.

GliomaPredict then performs a supervised PCA analysis using the PRINCOMP function as implemented in MATLAB Statistics Toolbox, which sequentially selects new orthogonal coordinates, capturing as much of the remaining variation in the data as possible (Figure 2). The PCA analysis effectively reduces the dimensionality of the data and the first three uncorrelated coordinates from the PCAview module were used to provide a visual representation of the subtypes, indicating the projection of the unknown sample in predefined subtypes. In addition, the output includes the prediction probability and distance of the unknown sample to the centroid of the O and G groups of reference samples. Depending on the initial assessment, the patient's profile is further stratified either into the OA/OB subtypes or into one out of four G subtypes using a specific set of subtype specific gene classifiers.

### Usability evaluation

To ensure that the tool performs consistently and reliably, we tested GliomaPredict on two levels. First, we set up a core test dataset consisting of 9 samples of new patients. The subtypes of these samples were assigned using PAMR, and results were cross-validated extensively using supervised hierarchical clustering and PCA to ensure the re-assignments of these samples to subtypes are consistent with the previous prediction. PAMR, which has a shrunken centroid algorithm implemented in [14], was originally used to derive the initial classifiers as reported by Li et al. [9]. Our initial test results indicated that the assignments of all the samples by the three methods were consistent. We then used these samples as testers to validate the performance consistency of GliomaPredict while introducing each of the main features or functions. For example, our original data were preprocessed using Windows version of dChip [15]. To comply with the Mac operating system and Linux platform, we chose to use a gdChip package for array normalization, which is an alternative version of dChip [16]. Our validation results using the core test set indicated that the data normalized using gdChip package perform consistently compared to those normalized by the original Windows dChip in terms of the subtype assignments. Secondly, we tested a subset of 61 samples with assigned subtypes that were predefined through an alternative approach using NMF and k-means stratification in our previous study [9]. GliomaPredict yielded a over 96% prediction accuracy when test samples were assigned to O/G main types, over 80% accuracy for predicting OA/OB subtypes and over 75% accuracy for predicting each of the four G subtypes (Table 1).

**Table 1:** Usability evaluation of glioma subtype predictions

Subtypes	Prediction accuracy (%)	
	
	**Train -> Test**^**§**^	**Test -> Test**^**#**^
O versus G	96	96

OA versus OB	78	80

4 G subtypes	60	75

^§^Train -> Test: Use classifiers from train set, classifier expression from train set to predict subtypes of test set

^#^Test -> Test: Use classifiers from train set, classifier expression from test set to predict subtypes of test set

### Current utilization

GliomaPredict is currently running on the server of the Neuro-Oncology Branch (NOB) at the National Institutes of Health (NIH). Physicians and clinicians routinely use the tool to predict the subtype of glioma patients. All newly generated expression .cel files are automatically uploaded to the server thereby making them readily available for analysis by the tool. Both clinicians and clinical/translational glioma researchers use the software to predict the subtypes of a given patient for the purpose of establishing the operative molecular pathways unique to that specific glioma subtype and for the purpose of identifying cohorts of patients most likely to respond to molecularly targeted agents based on our biological annotation of each subgroup.

## Discussion & Conclusions

We have previously reported on the creation of a glioma classification system that includes six hierarchically nested subtypes and six sets of classifiers to define each subtype. To make this classification schema clinically useful, we implemented GliomaPredict that utilizes gene expression profiles to reliably and automatically assign a glioma patient to one of the six predefined glioma subtypes. The tool is based on a supervised PCA analysis using classifiers that were derived previously and were extensively tested and validated. We have demonstrated that GliomaPredict performs reliably in assigning a new patient to one of the six glioma subtypes with a mere click of a button on any desktop computer connected to the server. Since the reference set of samples and subtype-specific gene classifiers can be taken from any given laboratory, we are confident that GliomaPredict will perform reliably across different laboratories and institutions.

More sophisticated computational methodologies and strategies will continue to be developed to help interpret and elucidate the underlying biology from the ever-growing body of "-omic" data being generated in gliomas and other tumor types. Nevertheless, there is a pressing need for user-friendly tools for computational naïve clinical and translational scientists if we are to begin to translate the advances in molecular and computational biology to the clinic and to patients. The ability of a clinical scientist to readily designate the molecular subtype of a particular patient's tumor will allow the design of clinical trials that may enrich for patients more likely to benefit from a novel molecularly targeted therapeutic agent based on the signal transduction pathway activation status inherent in that tumor subgroup. It is important to note, however, that the clinical utility of glioma molecular subtyping has yet to be demonstrated. Nevertheless, it is hypothesized that this type of tumor-specific molecular data will ultimately lead to more accurate patient prognostication and possibly even patient-specific therapy. The ready ability to assign a given patient's tumor to a particular subgroup by the simple click of a button on a clinical computer, will aid clinical scientists in the testing of that hypothesis. Thus, GliomaPredict represents a prototype of the kinds of clinically useful computational tools that may one day allow us to translate our rapidly growing knowledge of molecular and genetic biology toward the clinic and achieve the paradigm of personalized medicine.

## Availability and Requirements

Project name: GliomaPredict

Project home page: GliomaPredict is available from this site: https://wiki.nci.nih.gov/display/NOBbioinf/GliomaPredict

Operating system: Linux, Mac and Window OS

Requirements: MATLAB version 7.9.0 or higher, Perl version 5.8.8 or higher

Licence: a free, open source tool.

## Competing interests

The authors declare that they have no competing interests.

## Authors' contributions

AL, designed and developed the system, and drafted the manuscript. SB designed and developed the algorithm for reliability assessment and developed the glioma product web interface. SB and YK handled the GenePattern integration of the tool. HAF conceived, supervised and guided the project and assisted in the writing of the manuscript. All authors read and approved the final manuscript.

## Pre-publication history

The pre-publication history for this paper can be accessed here:

http://www.biomedcentral.com/1472-6947/10/38/prepub

## Supplementary Material

Additional file 1 **GliomaPredict Document**. The document in .pdf format described all the features in GliomaPredict and user information. Click here for file

Additional file 2 **GliomaPredict Installation**. The document in .pdf format provides the GliomaPredict installation instruction. Click here for file

Additional file 3 **GenePattern_Matlab_Windows_issue**. The document in .pdf format summarized the potential issues involved in using GenePattern and MATLAB associated with GliomaPredict tool and resolving solutions. Click here for file
