# Supplementary material for: GliomaPredict: a clinically useful tool for assigning glioma patients to specific molecular subtypes
Source: BMC Med Inform Decis Mak. 2010 Jul 15;10:38. doi: 10.1186/1472-6947-10-38 (PMC2912783; doi:10.1186/1472-6947-10-38)
Supplement: Additional file 1 — GliomaPredict Document. The document in .pdf format described all the features in GliomaPredict and user information. [file 1472-6947-10-38-S1.PDF]

GliomaPredict Documentation

Module name: GliomaPredict

Description: GliomaPredict is a web-based application that utilizes gene expression profiles and automatically assigns individual glioma patients to one of six predefined molecular subtypes reliably. The output of GliomaPredict includes subtype assignment represented graphically in a PCA view along with statistical measures that quantify the prediction accuracy.

Authors: Aiguo Li (liai@mail.nih.gov), Serdar Bozdag (bozdags@mail.nih.gov)
Neuro-Oncology Branch (NOB), NCI, NIH, Bethesda, MD

References

- GliomaPredict: a clinically useful tool for assigning glioma patients to specific molecular subtypes manuscript submitted to BMC Medical Informatics and Decision Making
- The module, installation documentation, additional files and sample data can be downloaded from the project's site: https://wiki.nci.nih.gov/display/NOBbioinf/GliomaPredict
- The glioma classification schema and the reference generation is described in the following archicle: Li, A., et al., *Unsupervised analysis of transcriptomic profiles reveals six glioma subtypes.* Cancer Res, 2009. **69**(5): p. 2091-9.

Requirements

- GenePattern: server version 3.0 or higher
- Matlab 2007a or later with Statistics toolbox installed on the server side

Input

- SampleID – name of the text file with expression data, located on the same machine where GenePattern installed. See "Data Format and Location" section for further information.

Output

The output of GliomaPredict is a self-contained printable PDF-file that contains four panels of PCA views representing six glioma subtypes with the unknow sample projected in PCA view. In addition, statistical measure of prediction probabilities and distances of unknown sample to the centroid of each subtyps are provided in the output (Figure 1).

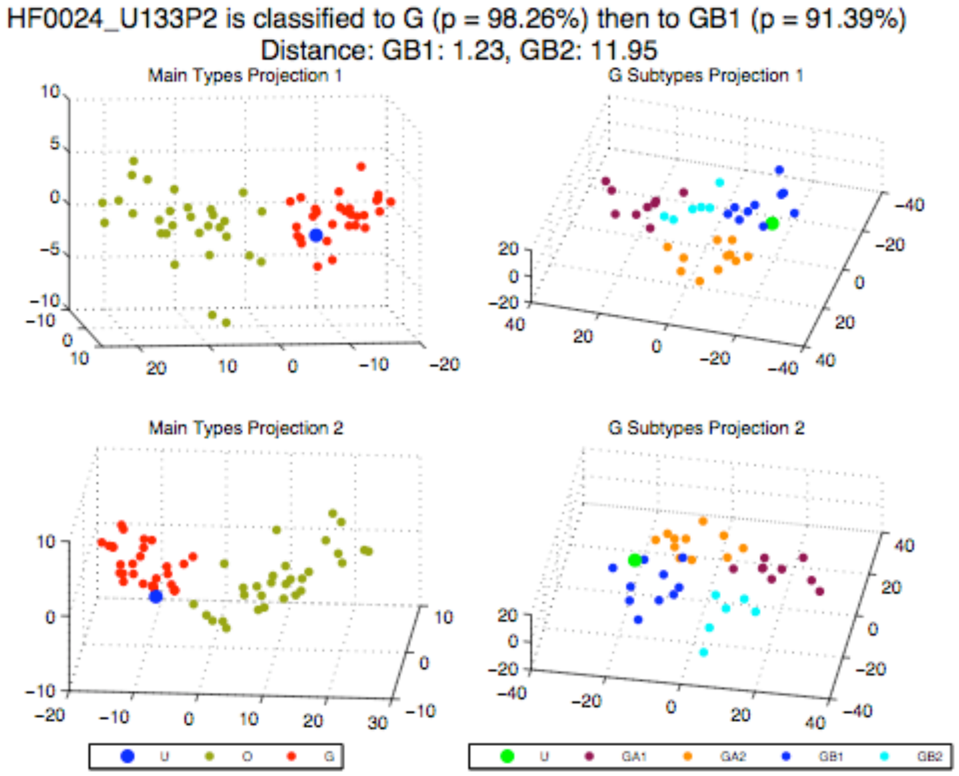

Figure 1. an example of GliomaPredict output

Data Format and Location

Data files

Data files stored by default in /common/GliomaPredict/formatted/GliomaPredict_dt/ directory (or C:\common\GliomaPredict\formatted\GliomaPredict_dt\ on Windows server).
Data files are normalized expression text files with SampleID as the file name with .txt extension. They have two or more columns: Probe set and normalized expression value. Use the file name as the header for second column. You can find data file examples in GLiomaPredict test data at https://wiki.nci.nih.gov/display/NOBbioinf/GliomaPredict.

Reference file

Reference file named reference.mat and located in the same directory as data files. It is binary MATLAB data file with DT structure that contains the following fields representing classifier probesets, classifier expression of O/G, OA/OB and GA1/GA2/GB1/GB2 subtypes and the subtype classes following the name schema below:
DT.OG54_dist_dt,
DT.OG54_dist_pids,
DT.OG54_dist_samples, (optional)
DT.OG54_dist_classes,
DT.OG54_pca_dt,
DT.OG54_pca_pids,
DT.OG54_pca_samples, (optional)
DT.OG54_pca_classes,
DT.OAOB69_dist_dt,
DT.OAOB69_dist_pids,
DT.OAOB69_dist_samples, (optional)
DT.OAOB69_dist_classes,
DT.OAOB69_pca_dt,

DT.OAOB69_pca_pids,
DT.OAOB69_pca_samples, (optional)
DT.OAOB69_pca_classes,
DT.G4_352_dist_dt,
DT.G4_352_dist_pids,
DT.G4_352_dist_samples, (optional)
DT.G4_352_dist_classes,
DT.G4_352_pca_dt,
DT.G4_352_pca_pids,
DT.G4_352_pca_samples, (optional)
DT.G4_352_pca_classes
