# Supplementary material for: GliomaPredict: a clinically useful tool for assigning glioma patients to specific molecular subtypes
Source: BMC Med Inform Decis Mak. 2010 Jul 15;10:38. doi: 10.1186/1472-6947-10-38 (PMC2912783; doi:10.1186/1472-6947-10-38)
Supplement: Additional file 2 — GliomaPredict Installation. The document in .pdf format provides the GliomaPredict installation instruction. [file 1472-6947-10-38-S2.PDF]

GliomaPredict Installation Documentation

GliomaPredict is a GenePattern module and is easy to install once you have GenePattern installed on server. However to function properly current design of the module requires additional files in certain location outside of GenePattern, so you will need administrator's access to the server to install those files. The figure 1 below illustrates the steps and workflow for GliomaPredict installation.

Please follow the step to install GliomaPredict:

1. Make sure GenePattern server version 3.0 or higher is installed properly and you have access to it and permission to add new modules. If GenePattern is not installed, download the package according to your platform from http://www.broad.mit.edu/cancer/software/genepattern/download/ and follow instructions how to install and configure the server.

2. Install GliomaPredict module.
   You can do it by 2 ways.
   a. Download GliomaPredict from NCI wiki website: https://wiki.nci.nih.gov/display/NOBbioinf/GliomaPredict
   b. In GenePattern go to ***Modules & Pipelines***, ***Install from zip***. Browse for the downloaded GliomaPredict.zip, select for which users to install it and press ***Install***.
   c. You can go to the same dialog without downloading the file. Paste into **URL of a GenePattern zip file** the following ULR: http://wiki.nci.nih.gov/display/NOBbioinf/GliomaPredict/GliomaPredict.zip

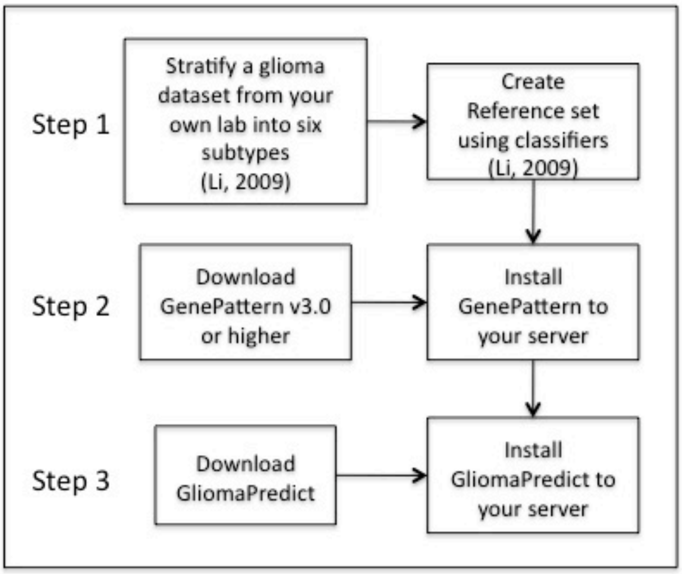

Figure 1 GliomaPredict installation flowchart.

3. Download GP reference_data.zip from http://wiki.nci.nih.gov/display/NOBbioinf/GliomaPredict/GP_reference data.zip.
4. Download GP_test_data.zip from http://wiki.nci.nih.gov/displayNOBbioinf/GP_test data.zip.
5. Unzip both zip files into C:\common\GliomaPredict\formatted\GliomaPredict_dt\ on Windows, or into /common/GliomaPredict/formatted/GliomaPredict_dt/ on Mac and

Linux.

6. If you'd like to have data files in another directory or under different name, you will have to edit PredictSubtypes.m in <GenePatternServer>/taskLib/GliomaPredict.x.xx/ directory. On Mac/Unix you may need to use sudo.

7. You can find documentation for the GliomaPredict module if you click HELP in the upper right corner of the corresponding GenePattern interface.

If you have any problems, questions or comments please contact Aiguo Li at liai@mail.nih.gov.

Thanks you for interest in our tool.

April 2010
