# Supplementary material for: GliomaPredict: a clinically useful tool for assigning glioma patients to specific molecular subtypes
Source: BMC Med Inform Decis Mak. 2010 Jul 15;10:38. doi: 10.1186/1472-6947-10-38 (PMC2912783; doi:10.1186/1472-6947-10-38)
Supplement: Additional file 3 — GenePattern_Matlab_Windows_issue. The document in .pdf format summarized the potential issues involved in using GenePattern and MATLAB associated with GliomaPredict tool and resolving solutions. [file 1472-6947-10-38-S3.PDF]

GenePattern: MATLAB issue on MS Windows based server

Summary

For GenePattern server installed on Windows, modules written in MATLAB show only startup.m file as an output, but it actually does not exist and clicking on the file link returns Tomcat error. The actual output files exist in GenePattern's jobResults directory, but they do not show up in GenePattern interface.

Description

On Windows MATLAB executed by a small matlab.exe file in <matlabrootfolder>\bin. This file detects operation system and then runs the real MATLAB executable, for example, in <matlabrootfolder>\bin\win32. Java wrapper that executes Matlab on GenePattern considers only the first matlab.exe as a process it supposes to run, and when it terminates, the whole module is terminated and generates the output reports. The report at this point contains only matlab script startup.m. However, matlab is still running, but GenePattern does not know anything about it. When MATLAB script is about to end, it deletes startup.m, this is why clicking on this file from GenePattern generates error.

Workaround

The workaround would to make Java wrapper to execute the real matalab.exe. There are several ways to do it.

1. There are should be two directories in PATH environment variable: <matlabrootfolder>\bin and <matlabrootfolder>\bin\win32. Put the win32 before the bin. Reboot the machine.
2. Or rename files matlab.exe and matlab.bat in <matlabrootfolder>\bin. Make all shortcuts to target <matlabrootfolder>\bin\win32\matlab.exe
3. Change the Java code of RunMatlab class to set full path to platform-specific matlab.exe. The code will be provided on request.

Any other solution/workarounds are welcome.
